# Evaluation of accuracy of photogrammetry with 3D scanning and conventional impression method for craniomaxillofacial defects using a software analysis

**DOI:** 10.1186/s13063-022-07005-1

**Published:** 2022-12-27

**Authors:** Arushi Beri, Sweta Kale Pisulkar, Ashutosh D. Bagde, Akansha Bansod, Chinmayee Dahihandekar, Balaji Paikrao

**Affiliations:** 1 Department of Prosthodontics, Sharad Pawar Dental College and Hospital, Datta Meghe Institute of Higher Education and Research, Wardha, India; 2 Faculty of Engineering and Technology, Datta Meghe Institute of Higher Education and Research, Wardha, India; 3 Datta Meghe Institute of Higher Education and Research, Wardha, India

**Keywords:** Photogrammetry, 3D scanning, Conventional impression method, Craniomaxillofacial defects

## Abstract

**Background:**

Facial mutilation and deformities can be caused by cancer, tumours, injuries, infections, and inherited or acquired deformities and has the potential to degrade one’s quality of life by interfering with fundamental tasks like communication, breathing, feeding, and aesthetics.

Depending on the type of defect, producing maxillofacial prostheses for the rehabilitation of patients with various defects can be challenging and complex. The prosthesis is used to replace missing or damaged parts of the cranium and face, like the nose, auricle, orbit, and surrounding tissues, as well as missing areas of soft and hard tissue, with the primary goal of increasing the patient’s quality of life by rehabilitating oral functions such as speech, swallowing, and mastication.

Traditional maxillofacial prosthesis impression and fabrication processes include a number of complicated steps that are costly, time-consuming, and uncomfortable for the patient. These rely on the knowledge of the maxillofacial team, dental clinicians, and maxillofacial technician.

The foundation of the impression is the keystone for creating a prosthesis. However, this is the most time-consuming and difficult chair-side operation in maxillofacial prosthesis manufacturing since it requires prolonged interaction with the patient. The field of prosthesis fabrication is being transformed by the digital revolution. Digital technology allows for more accurate impression data to be gathered in less time (3 to 5 min) than traditional methods, lowering patient anxiety. Digital impressions eliminate the need for messy impression materials and provide patients with a more pleasant experience. This method bypasses the procedure of traditional gypsum model fabrication. This eliminates the disparity caused by a dimensional distortion of the impression material and gypsum setting expansion. Traditional dental impression processes leave enough room for errors, such as voids or flaws, air bubbles, or deformities, while current technology for prosthesis planning has emerged as an alternative means to improve patient acceptability and pleasure, not only because the end result is a precisely fitted restoration but also because the chair-side adjustments required are reduced.

The most frequent approaches for creating 3D virtual models are the following. To begin, 3D scanning is employed, in which the subjects are scanned in three dimensions, and the point cloud data is used to create a virtual digital model.

**Methods:**

It will be a hospital-based randomised control trial, carried out at the Department of Prosthodontics, Sharad Pawar Dental College, Sawangi (Meghe), Wardha, a part of Datta Meghe Institute of Medical Sciences (Deemed University). A total of 45 patients will be selected from the outpatient department (OPD) of the Department of Prosthodontics. All the patients will be provided written consent before their participation in the study.

**Methodology:**

1. Patient screening will be done, and the patient will be allocated to three techniques that are the conventional manual method, photogrammetry method, and 3D scanning in a randomised manner

2. The impression of the defect will be recorded by conventional manual method, photogrammetry method, and 3D scanning

3. The defect will be modelled in three ways: first is as per the manual dimension taken on the patient, second is the organisation of photographic image taken with lab standards and third is plotting of point cloud data to generate the virtual 3D model

4. For photogrammetric prosthesis design, finite photos/images will be taken at multiple angles to model the 3D virtual design. With the use of minimum photographs, the 3D modelling can be performed by using freeware, and a mould is obtained

5. The CAD software was used to design the prosthesis, and the final negative mould can be printed using additive manufacturing

6. The mould fabricated by all three methods will be analysed by a software using reverse engineering technology

**Study design**: Randomised control trial

**Duration**: 2 years

**Sample size**: 45 patients

**Discussion:**

Rodrigo Salazar-Gamarra1, Rosemary Seelaus, and Jorge Vicente Lopes da Silva et al., in the year 2016, discussed, as part of a method for manufacturing face prostheses utilising a mobile device, free software, and a photo capture protocol, that 2D captures of the anatomy of a patient with a facial defect were converted into a 3D model using monoscopic photogrammetry and a mobile device. The visual and technical integrity of the resulting digital models was assessed. The technological approach and models that resulted were thoroughly explained and evaluated for technical and clinical value.

Marta Revilla-León, Wael Att, and Dr Med Dent et al. (2020) used a coordinate measuring equipment which was used to assess the accuracy of complete arch implant impression processes utilising conventional, photogrammetry, and intraoral scanning.

Corina Marilena Cristache and Ioana Tudor Liliana Moraru et al. in the year 2021 provided an update on defect data acquisition, editing, and design using open-source and commercially available software in digital workflow in maxillofacial prosthodontics.

This research looked at randomised clinical trials, case reports, case series, technical comments, letters to the editor, and reviews involving humans that were written in English and included detailed information on data acquisition, data processing software, and maxillofacial prosthetic part design.

**Trial registration:**

CTRI/2022/08/044524. Registered on September 16, 2022

**Supplementary Information:**

The online version contains supplementary material available at 10.1186/s13063-022-07005-1.

## Introduction

### Background and rationale {6a}

#### Background

Facial mutilation and deformities can be caused by cancer, tumours, injuries, infections, and inherited or acquired deformities and has the potential to degrade one’s quality of life by interfering with fundamental tasks like communication, breathing, feeding, and aesthetics.

Depending on the type of defect, producing maxillofacial prostheses for the rehabilitation of patients with various defects can be challenging and complex. The prosthesis is used to replace missing or damaged parts of the cranium and face, like the nose, auricle, orbit, and surrounding tissues, as well as missing areas of soft and hard tissue, with the primary goal of increasing the patient’s quality of life by rehabilitating oral functions such as speech, swallowing, and mastication.

Traditional maxillofacial prosthesis impression and fabrication processes include a number of complicated steps that are costly, time-consuming, and uncomfortable for the patient. These rely on the knowledge of the maxillofacial team, dental clinicians, and maxillofacial technician [1].

The foundation of the impression is the keystone for creating a prosthesis. However, this is the most time-consuming and difficult chair-side operation in maxillofacial prosthesis manufacturing since it requires prolonged interaction with the patient. The field of prosthesis fabrication is being transformed by the digital revolution. Digital technology allows for more accurate impression data to be gathered in less time (3 to 5 min) than traditional methods, lowering patient anxiety. Digital impressions eliminate the need for messy impression materials and provide patients with a more pleasant experience [1]. This method bypasses the procedure of traditional gypsum model fabrication. This eliminates the disparity caused by a dimensional distortion of the impression material and gypsum setting expansion. Traditional dental impression processes leave enough room for errors, such as voids or flaws, air bubbles, or deformities. While current technology for prosthesis planning has emerged as an alternative means to improve patient acceptability and pleasure, not only because the end result is a precisely fitted restoration but also because the chair-side adjustments required are reduced [2].

#### Rationale

The most frequent approaches for creating 3D virtual models are the following. To begin, 3D scanning is employed, in which the subjects are scanned in three dimensions, and the point cloud data is used to create a virtual digital model. The most difficult and time-consuming procedure in maxillofacial prosthesis is making an impression of the abnormality. Digital scanning is vital for achieving minimal interaction with the patient, and it avoids manual manufacture of the cast, resulting in no dimensional deformation of the cast.

In this COVID era, less interaction between patient and physician means a reduced danger of disease transmission. The photogrammetric method can be utilised as a first step toward obtaining a precise 3D representation of a defect that is acceptable for low-cost implementation.

### Objectives {7}

1. To assess and evaluate the conventional method of craniomaxillofacial impression procedures
2. To assess and evaluate the photogrammetric method of craniomaxillofacial impression procedures
3. To assess and evaluate the 3D scanning method of craniomaxillofacial impression procedures
4. To compare the conventional, photogrammetry, and extraoral 3D scanning accuracy of craniofacial and midfacial impression procedures evaluated with a software

### Trial design {8}

The randomised control trial will be conducted at the Department of Prosthodontic, Sharad Pawar Dental College, in an isolated setup. The study protocol will be explained to the participating patients. Furthermore, a written informed consent will be obtained from their parents**.**

## Methods: participants, interventions and outcomes

### Study setting {9}

The study will be conducted at the Department of Prosthodontics Crown and Bridge Sharad Pawar Dental College and Hospital DMIMSU Sawangi Meghe Wardha.

Methodology:

1. Patient screening will be done and the patient will be allocated to three techniques that are conventional manual method, photogrammetry method, and 3D scanning in a randomised manner
2. The impression of the defect will be recorded by conventional manual method, photogrammetry method, and 3D scanning
3. The defect will be modelled in three ways: first is as per the manual dimension taken on patient, second is the organisation of photographic image taken with lab standards and third is plotting of point cloud data to generate the virtual 3D model
4. For Photogrammetric prosthesis design: Finite photos/images will be taken at multiple angles to model the 3D virtual design. With the use of minimum photographs, the 3D modelling can be performed by using freeware, and a mould is obtained
5. The CAD software was used to design the prosthesis, and the final negative mould can be printed using additive manufacturing
6. The mould fabricated by all three methods will be analysed by a software using reverse engineering technology

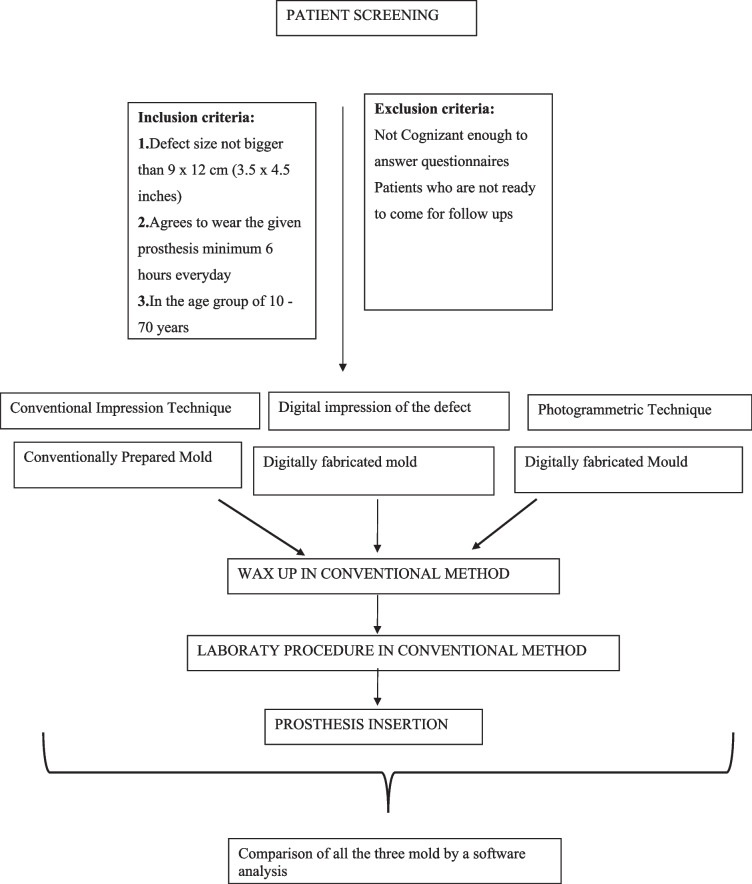


### Eligibility criteria {10}

- Defect size not bigger than 9 × 12 cm (3.5 × 4.5 inches)
- In the age group of 10–70 years
- Extra oral defects

### Who will take informed consent? {26a}

Informed consent or assent from potential trial participants or authorised surrogates will be taken by the primary investigator

### Additional consent provisions for collection and use of participant data and biological specimens {26b}

Additional consent provisions for collection and use of participant data and biological specimens in ancillary studies will be taken by the primary investigator.

## Interventions

### Explanation for the choice of comparators {6b}

Photogrammetry will be evaluated with 3D scanning and conventional impression method for craniomaxillofacial defects using a software analysis.

### Intervention description {11a}

#### Introduction

Facial mutilation and deformities can be caused by cancer, tumours, injuries, infections, and inherited or acquired deformities and has the potential to degrade one’s quality of life by interfering with fundamental tasks like communication, breathing, feeding, and aesthetics.

Depending on the type of defect, producing maxillofacial prostheses for the rehabilitation of patients with various defects can be challenging and complex. The prosthesis is used to replace missing or damaged parts of the cranium and face, like the nose, auricle, orbit, and surrounding tissues, as well as missing areas of soft and hard tissue, with the primary goal of increasing the patient's quality of life by rehabilitating oral functions such as speech, swallowing, and mastication [3].

Traditional maxillofacial prosthesis impression and fabrication processes include a number of complicated steps that are costly, time-consuming, and uncomfortable for the patient. These rely on the knowledge of the maxillofacial team, dental clinicians, and maxillofacial technician [1].

The foundation of the impression is the keystone for creating a prosthesis. However, this is the most time-consuming and difficult chair-side operation in maxillofacial prosthesis manufacturing since it requires prolonged interaction with the patient. The field of prosthesis fabrication is being transformed by the digital revolution. Digital technology allows for more accurate impression data to be gathered in less time (3 to 5 min) than traditional methods, lowering patient anxiety. Digital impressions eliminate the need for messy impression materials and provide patients with a more pleasant experience [1]. This method by pass the procedure of traditional gypsum model fabrication. This eliminates the disparity caused by a dimensional distortion of the impression material and gypsum setting expansion. Traditional dental impression processes leave enough room for errors, such as voids or flaws, air bubbles, or deformities. While current technology for prosthesis planning has emerged as an alternative means to improve patient acceptability and pleasure, not only because the end result is a precisely fitted restoration but also because the chair-side adjustments required are reduced [2].

The most frequent approaches for creating 3D virtual models are the following. To begin, 3D scanning is employed, in which the subjects are scanned in three dimensions, and the point cloud data is used to create a virtual digital model.

Photogrammetry is the second method, which uses finite pictures taken from various perspectives to create 3D digital models. When compared to traditional data collection methods, the two methods are non-contact and save time. Though 3D scanning is a quick and accurate process, it has drawbacks such as a high set-up cost and the need for expert labour. Because of these drawbacks, this technology is only used in prosthetic design on a very limited basis. With a little compromise in 3D modelling accuracy, the photogrammetry method can overcome the drawbacks of 3D scanning. It primarily offers advantages such as the usage of freeware for model creation, the ability to handle the method with semi-skilled people, and the ability to produce good results even with a phone camera using standard operating procedures [4].

The initial step in the photogrammetry approach is to collect photos from all angles; after that, post-processing can be done by a professional using photogrammetry software and CAD/CAM tools. The photogrammetrically formed 3D model can then be used to create a mould, which can be compared against moulds fabricated via 3D scanning and traditional methods [5].

Because the prosthodontist may review the digitally scanned impression at the chairside just by magnifying it, any problems can be corrected before the data is sent to the dental laboratory [6]. However, the complete manufacture of the prosthesis using rapid prototyping is expensive, and the final prosthesis creation is fully dependent on the donor anatomy [7].

When compared to a manually modelled prosthesis, a 3D-produced prosthesis made from the mirror image of the undamaged portion or from the donor does not provide improved aesthetics. The art of sculpturing and adding uniqueness into the prosthesis can be added using the traditional wax-up procedure [8]. Other laboratory procedures can be done using the traditional way to keep costs down and make the prosthesis more appealing [9–11]. 

The purpose of this research is to assess and compare conventional impression techniques, photogrammetry, and 3D scanning methods of impression making and casting by rapid prototyping for the manufacturing of maxillofacial prostheses with better precision and fit while reducing chair side time and minimising patient contact while keeping costs low.

### Criteria for discontinuing or modifying allocated interventions {11b}

There will be no special criteria for discontinuing or modifying allocated interventions

### Strategies to improve adherence to interventions {11c}

**Fig. 1:**
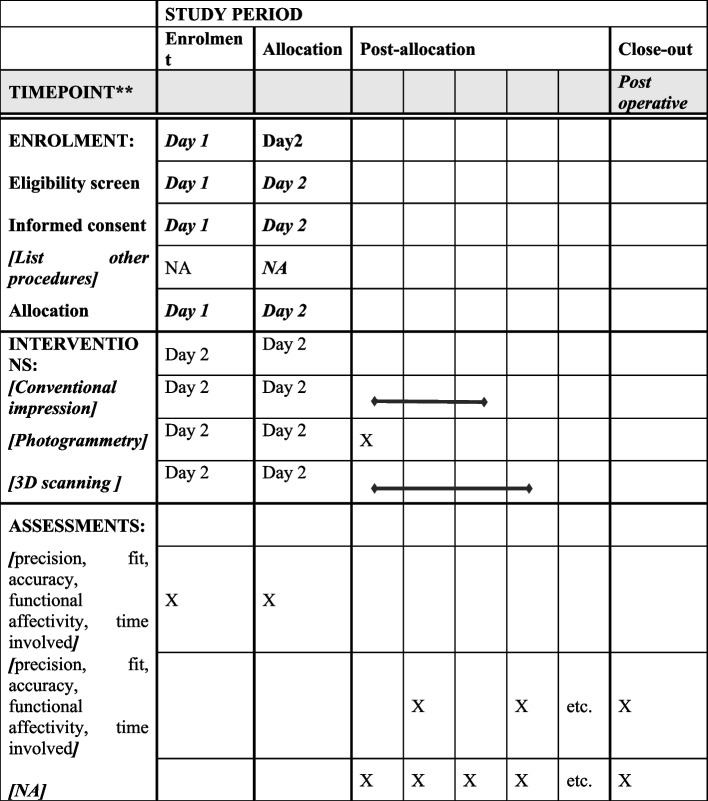
Study period

Not applicable as there are no follow-ups involved in the study. The outcomes will be checked on the day of prosthesis insertion therefore adherence to intervention will not be an issue because the patients will be evaluated for primary and secondary outcomes on the day of prosthesis insertion because cranioplasty is an elective surgical procedure carried out for the purpose of improving aesthetics and in some cases function. We are using PMMA flaps for cranioplasty procedures which have a complication rate of 9.2–23% (most commonly infection) according to a study by Enrique-Caro-Osiro et al. Further studies have shown a slight decrease in infection rate when performed within 3 months of craniectomy (Fig. 1).

### Relevant concomitant care permitted or prohibited during the trial {11d}

Not applicable because there is no concomitant care permitted or prohibited during the trial because cranioplasty is elective surgical procedure carried out for the purpose of improving aesthetics and in some cases function. We are using PMMA flaps for cranioplasty procedures which have a complication rate of 9.2–23% (most commonly infection) according to a study by Enrique-Caro-Osiro et al. Further studies have shown a slight decrease in infection rate when performed within 3 months of craniectomy.

### Provisions for post-trial care {30}

Not applicable as there is no follow-up involved in the study as cranioplasty is an elective surgical procedure carried out for the purpose of improving aesthetics and, in some cases, function. We are using PMMA flaps for cranioplasty procedures which have a complication rate of 9.2–23% (most commonly infection) according to a study by Enrique-Caro-Osiro et al. Further studies have shown a slight decrease in infection rate when performed within 3 months of craniectomy.

### Outcomes {12}

The study would help in predicting the best method for craniomaxillofacial impression procedures.

The final prosthesis will be evaluated for precision, fit, accuracy, functional affectivity, time involved in comparison with conventional, photogrammetry and 3D scanning technology. The low-cost setup of the photogrammetric method gives the advantage of minimum chair side time and less patient exposure which is very much advantageous in the COVID era.

### Participant timeline {13}

The participant timeline is as follows: 25 October 2022 to 25 August 2023.

### Sample size {14}

45 Daniel formula for sample size calculation

$$n=\frac{Za/{2}^2\cdot P\cdot \left(1-P\right)}{d^2}$$

where*Zα*/2^2^ is the level of significanceAt 5%, i.e. 95% confidence interval = 1.96*P* = prevalence of craniomaxillofacial defects = 3% = 0.03*d* = desired error of margin = 5% = 0.05

$$n=\frac{1.96^2\times 0.03\times \left(1-0.03\right)}{0.05^2}=44.71$$

*n* = 45 patients needed for the study

Study reference: Mohamed Khallaf et al.

Formula reference: Daniel et al.

Statistical method: chi-square test, one-way ANOVA, Tukey multiple comparison test

Software used: SPSS 27.0 V and Graph Pad Prism 7.0V

### Recruitment {15}

Patients in the OPD of Prosthodontic and Crown and Bridge Department of Sharad Pawar Dental College and Hospital DMIMSU Sawangi Meghe Wardha were recruited.

## Assignment of interventions: allocation

### Sequence generation {16a}

Computer-generated random numbers

### Concealment mechanism {16b}

On-site computer system

### Implementation {16c}

The co-investigator of the department will generate the allocation sequence, will enrol participants, and will assign participants to interventions.

## Assignment of interventions: blinding

### Who will be blinded {17a}

Trial participants, outcome assessors, and data analysts

### Procedure for unblinding if needed {17b}

Data analysis from spreadsheets of random numbers was done to determine the assignment; there is no human involvement; and the process is fully concealed from both study investigators and prospective participants until the study arm is assigned.

## Data collection and management

### Plans for assessment and collection of outcomes {18a}

Software analysis of all the prostheses fabricated by all three ways

### Plans to promote participant retention and complete follow-up {18b}

Not applicable as there are no follow-ups involved in the study. The outcomes will be checked on the day of prosthesis insertion; therefore, adherence to intervention will not be an issue because the patients will be evaluated for primary and secondary outcomes on the day of prosthesis insertion because cranioplasty is an elective surgical procedure carried out for the purpose of improving aesthetics and, in some cases, function. We are using PMMA flaps for cranioplasty procedures which have a complication rate of 9.2–23% (most commonly infection) according to a study by Enrique-Caro-Osiro et al. Further studies have shown a slight decrease in infection rate when performed within 3 months of craniectomy.

### Data management {19}

Electronic data management will be done; the primary investigator will collect the data and will enter the data into the database for screening and randomisation purposes.

### Confidentiality {27}

Personal information about potential and enrolled participants will be collected and maintained in order to protect confidentiality before, during, and after the trial.

### Plans for collection, laboratory evaluation and storage of biological specimens for genetic or molecular analysis in this trial/future use {33}

Not applicable as there is no collection, laboratory evaluation and storage of biological specimens for genetic or molecular analysis in this trial because the procedure of the trial is explained as follows

1. The impression of the defect will be recorded by Conventional manual method, photogrammetry method, and 3D scanning.
2. The defect will be modelled in three ways: first is as per the manual dimension taken on the patient, second is organisation of photographic image taken with lab standards and third is plotting of point cloud data to generate the virtual 3D model.
3. For photogrammetric prosthesis design: finite photos/images will be taken at multiple angles to model the 3D virtual design. With the use of minimum photographs, the 3D modelling can be performed by using freeware, and a mould is obtained.
4. The CAD software was used to design the prosthesis, and the final negative mould can be printed using additive manufacturing.
5. The mould fabricated by all three methods will be analysed by a software using reverse engineering technology

Clearly, biological specimens will not be collected, so this field is not applicable.

## Statistical methods

### Statistical methods for primary and secondary outcomes {20a}

Analytical tests like chi-square test and Student’s *t*-test will be performed. All the statistical analysis will be performed; *p* 0.05 will be considered as the level of significance.

### Interim analyses {21b}

Not applicable as we are inserting the prosthesis on the day of surgery, so interim analysis is not required; the outcomes will be checked on the day of prosthesis insertion; therefore, adherence to intervention will not be an issue because the patients will be evaluated for primary and secondary outcomes on the day of prosthesis insertion because cranioplasty is an elective surgical procedure carried out for the purpose of improving aesthetics and, in some cases, function.

### Methods for additional analyses (e.g. subgroup analyses) {20b}

#### SPIRIT guidance: methods for any additional analyses (e.g. subgroup and adjusted analyses)

As there are no subgroups in the study, the subgroup analysis will not be carried out. The population will be studied under three groups—conventional impression, 3D scanning, and photogrammetry—so there is no role of subgroups.

### Methods in analysis to handle protocol non-adherence and any statistical methods to handle missing data {20c}

NA

As noted above in the additional analysis, a secondary, per-protocol analysis will examine outcomes by whether or not the participant was engaged in shared decision-making, as reported by clinicians and judged by study staff, regardless of whether the decision aid was used.

Missing data will be described, and, if extensive, limitations will be identified in our discussion. We will compute standardised effect sizes to help determine the degree of imbalance on baseline characteristics and subsequently inform multivariable model development.

### Plans to give access to the full protocol, participant level-data and statistical code {31c}

The datasets analysed during the current study and statistical code are available from the corresponding author on reasonable request, as is the full protocol.

## Oversight and monitoring

### Composition of the coordinating Centre and trial steering committee {5d}

The committee consist of the following:

1. The head of the department of prosthodontics
2. P.G. guide
3. Research scientist
4. Principal investigator
5. Surgeon
6. Statistician
7. Data manager

### Composition of the data monitoring committee, its role and reporting structure {21a}

Composition of data monitoring committee (DMC) is as follows: research in charge of the institute and research in charge of the department.

### Adverse event reporting and harms {22}

Data will be collected, assessed, and spontaneously reported during adverse events and other unintended effects of trial interventions or trial conduct.

### Frequency and plans for auditing trial conduct {23}

The project management group meet will review the trial conducted every month. The trial steering group and the independent data monitoring and ethics committee meet will review and conduct the trial period till the trial is complete.

### Plans for communicating important protocol amendments to relevant parties (e.g. trial participants, ethical committees) {25}

The sponsor and funder will be notified first; then, the PI will notify the centres, and a copy of the revised protocol will be sent to the PI to add to the Investigator Site File.

### Dissemination plans {31a}

Publication

## Trial status

[ref no: DMIMSU(DU)/IEC/2022/781] protocol version number 1; recruitment began on September 2022 and approximately will be completed by January 2024.

## Supplementary Information

**Additional file 1.**

## Data Availability

Access to investigator
