# Supplementary figures and images for: Evaluation of accuracy of photogrammetry with 3D scanning and conventional impression method for craniomaxillofacial defects using a software analysis
Source: Trials. 2022 Dec 27;23:1048. doi: 10.1186/s13063-022-07005-1 (PMC9793656; doi:10.1186/s13063-022-07005-1)

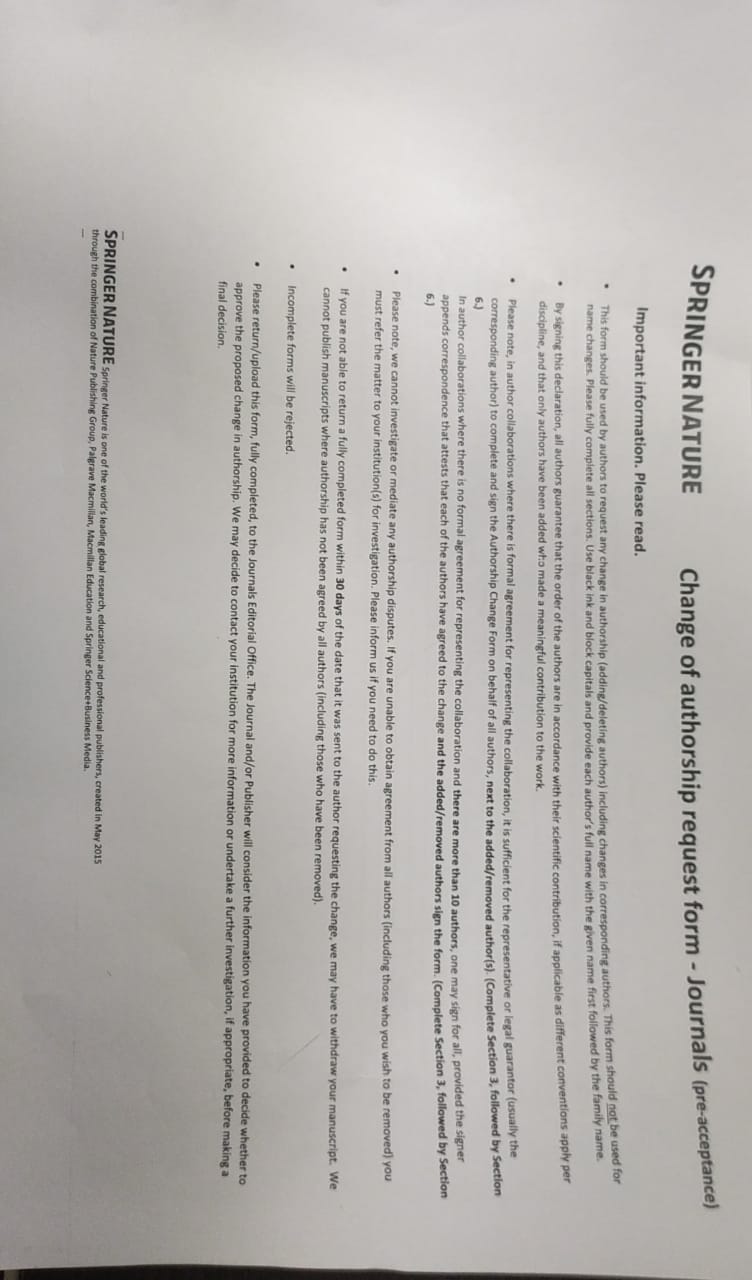


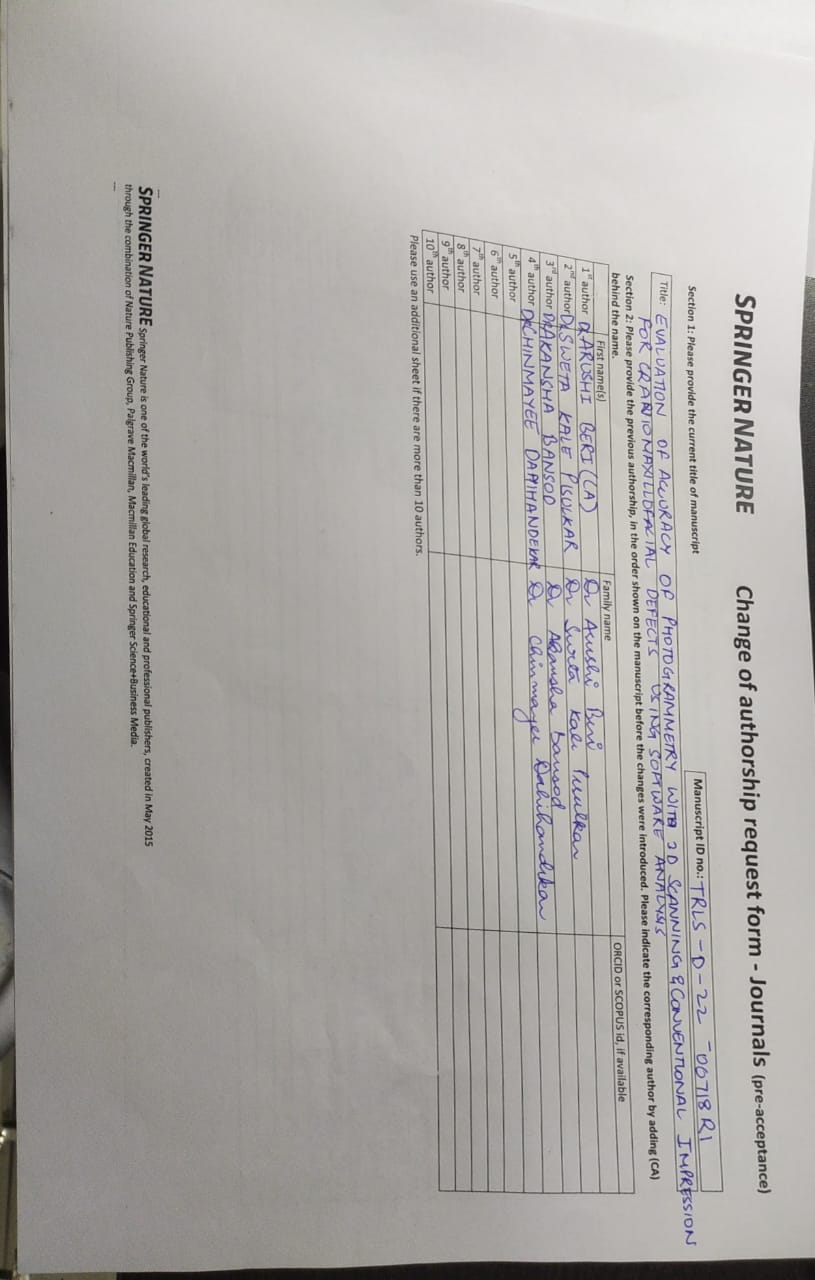


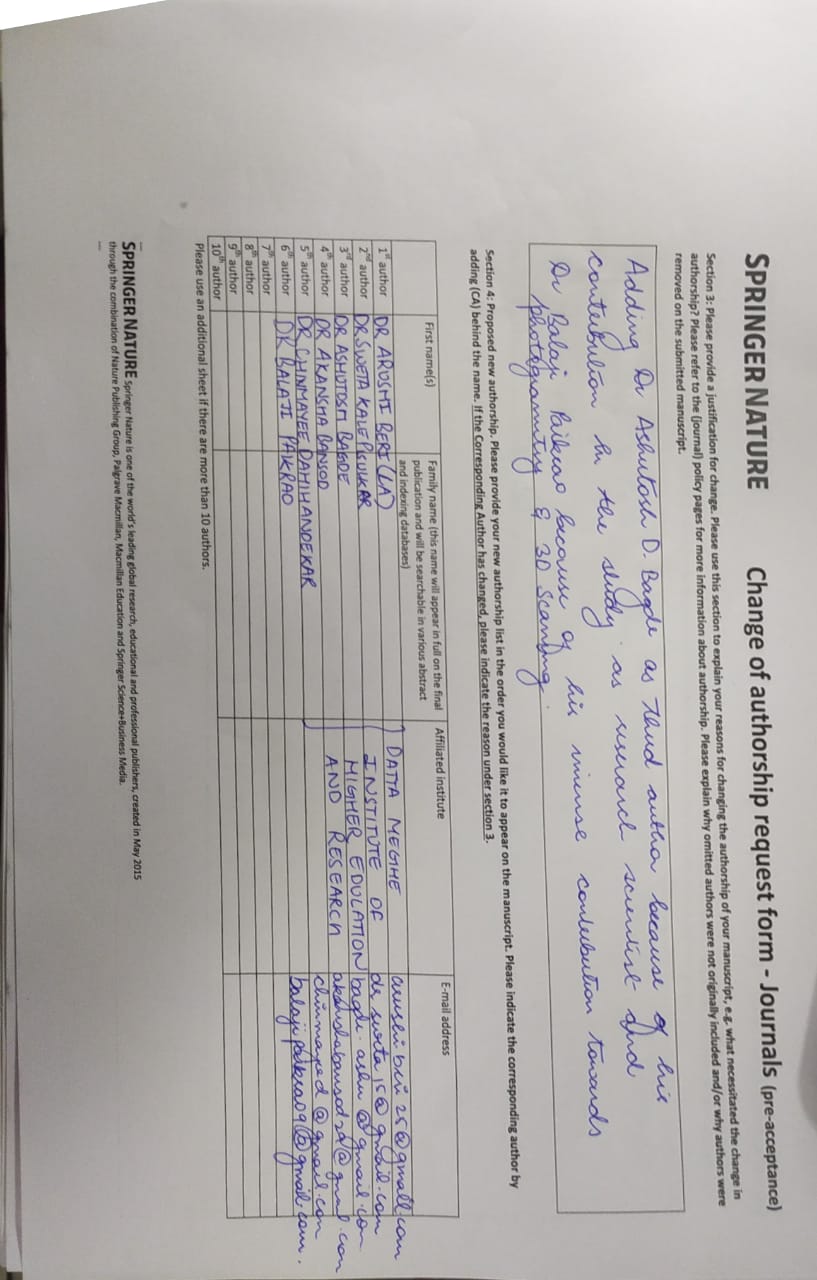


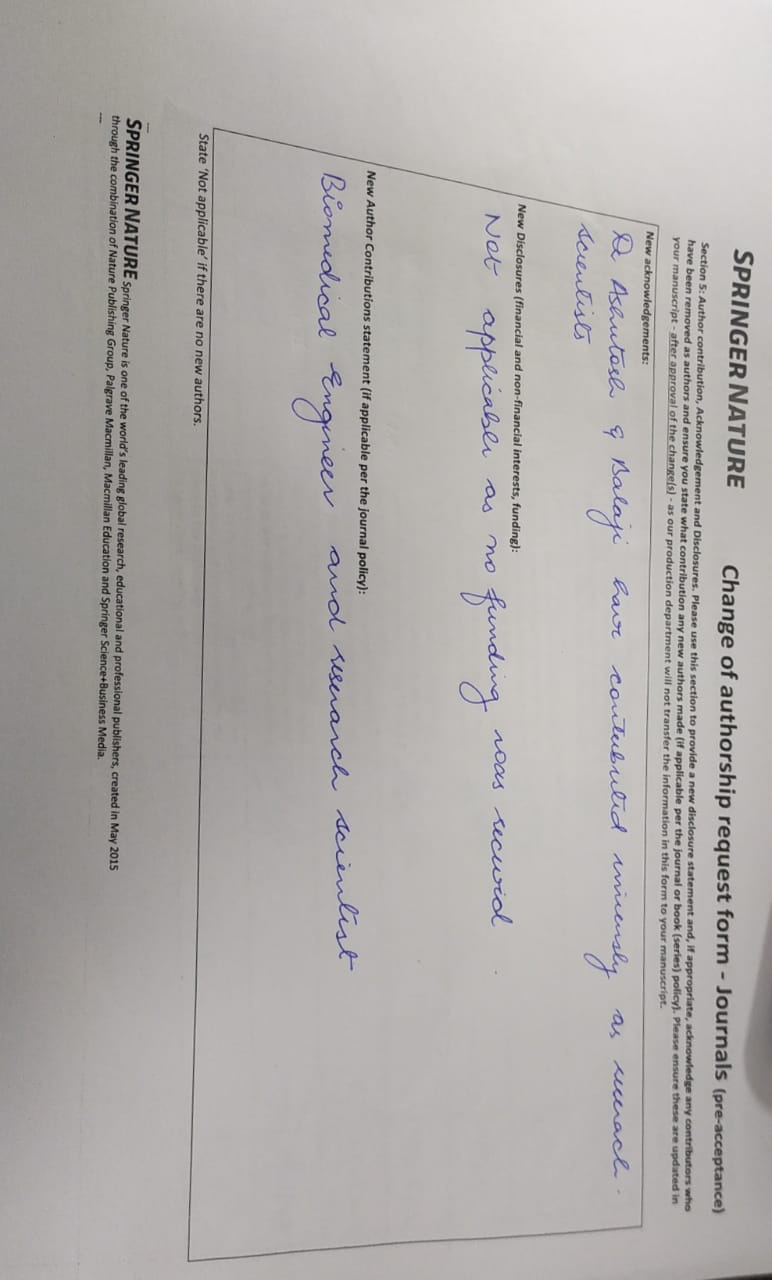

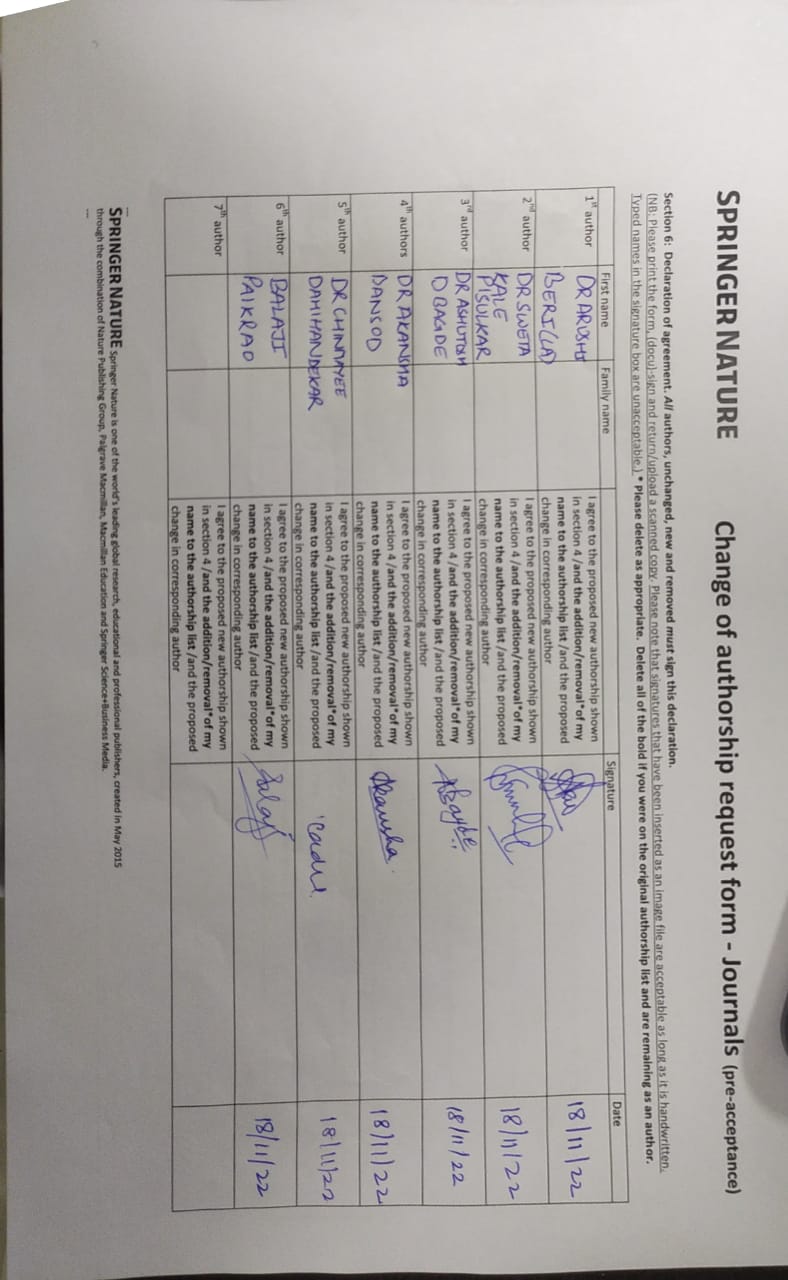

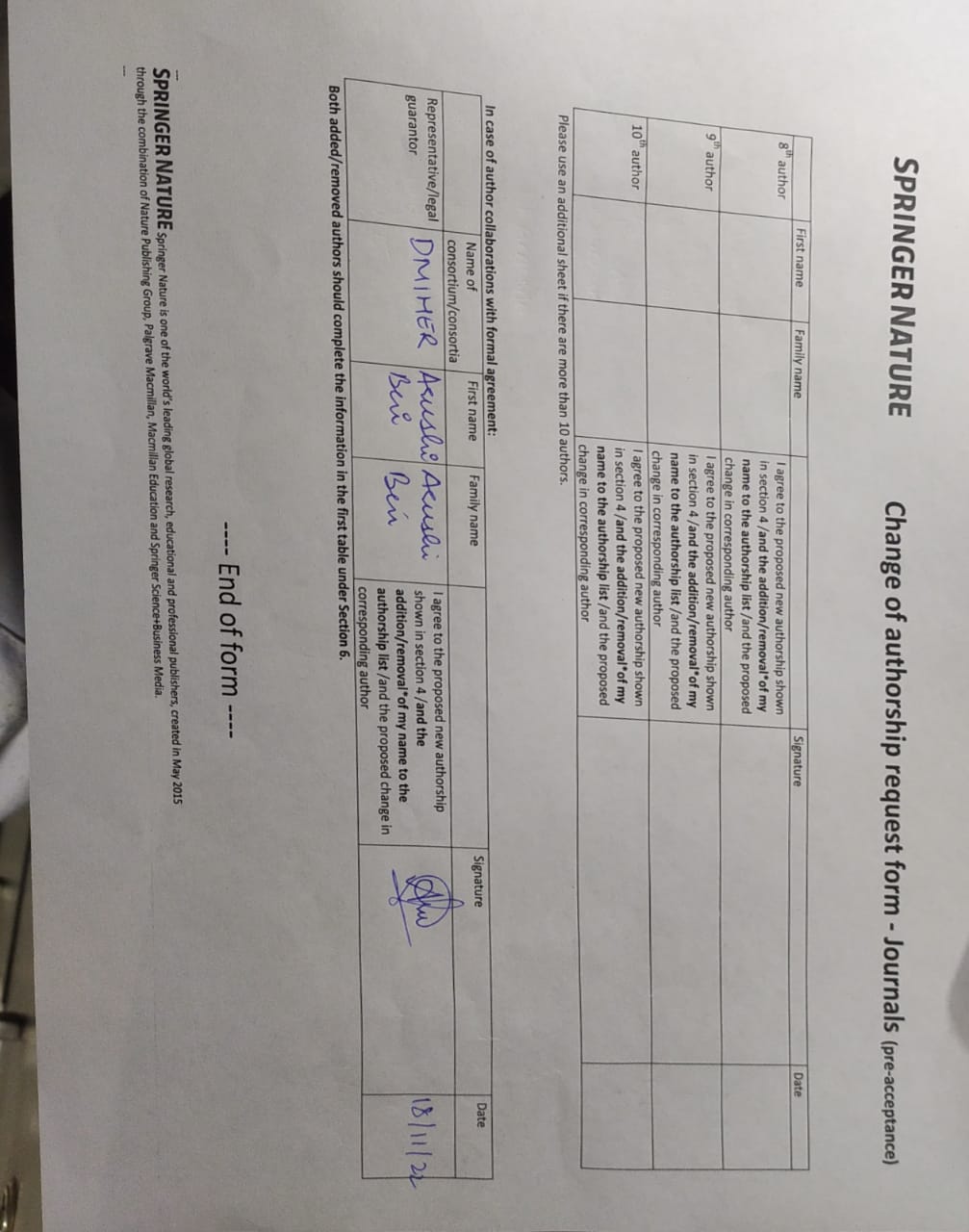

Supplement: Supplementary file 1 — Additional file 1. [file 13063_2022_7005_MOESM1_ESM.docx]
